# Impact of sodium–glucose cotransporter 2 inhibitors on renal function in participants with type 2 diabetes and chronic kidney disease with normoalbuminuria

**DOI:** 10.1186/s13098-020-0516-9

**Published:** 2020-01-10

**Authors:** Akinobu Nakamura, Hideaki Miyoshi, Hiraku Kameda, Kumiko Yamashita, Yoshio Kurihara

**Affiliations:** 1 Department of Rheumatology, Endocrinology and Nephrology, Faculty of Medicine and Graduate School of Medicine, Hokkaido University Graduate School of Medicine, N-15, W-7, Kita-ku, Sapporo, 060-8638 Japan; 2 Division of Diabetes and Obesity, Faculty of Medicine and Graduate School of Medicine, Hokkaido University Graduate School of Medicine, Sapporo, Japan; 3 Kurihara Clinic, Sapporo, Japan

**Keywords:** Chronic kidney disease, Diabetic kidney disease, Estimated glomerular filtration rate, sodium–glucose cotransporter 2 inhibitor

## Abstract

**Background:**

We compared the effects of sodium–glucose cotransporter 2 (SGLT2) inhibitors on renal function in participants with type 2 diabetes and chronic kidney disease (CKD) classified by degree of albuminuria.

**Methods:**

A retrospective review of the clinical records of Japanese participants with type 2 diabetes (age > 20 years; SGLT2 inhibitor treatment > 2 years; estimated glomerular filtration rate (eGFR) < 60 mL/min/1.73 m^2^) was conducted. Based on the urinary albumin-to-creatinine ratio (UACR) or urinary protein-to-creatinine ratio (UPCR) at the start of SGLT2 inhibitor administration, participants were categorized into three groups: normoalbuminuria (A1; UACR < 30 mg/g Cr or UPCR < 0.15 g/g Cr), microalbuminuria (A2; UACR 30 to < 300 mg/g Cr or UPCR 0.15 to < 0.50 g/g Cr), and macroalbuminuria (A3; UACR ≥ 300 mg/g Cr or UPCR ≥ 0.50 g/g Cr). The study outcome was a comparison of the rates of change in renal function evaluated by eGFR at 2 years after starting SGLT2 inhibitor among the three groups.

**Results:**

A total of 87 participants (40 females, 47 males) were categorized into three groups: A1 (*n* = 46), A2 (*n* = 25), and A3 (*n* = 16). eGFR was similarly decreased at 2 years before starting SGLT2 inhibitor in all three groups. However, the decline in eGFR was ameliorated at 2 years after starting SGLT2 inhibitor, and eGFR was rather increased in the A1 and A2 groups. Interestingly, the rate of change in eGFR at 2 years after starting SGLT2 inhibitor in the A1 group was significantly higher than that in the A3 group.

**Conclusions:**

These results demonstrate that more favorable effects of SGLT2 inhibitors on renal function were observed in participants with type 2 diabetes and CKD with normoalbuminuria compared with those with macroalbuminuria.

*Trial registration* UMIN-CTR: UMIN000035263. Registered 15 December 2018

## Background

Large randomized clinical trials have indicated that sodium–glucose cotransporter 2 (SGLT2) inhibitors can significantly ameliorate renal outcomes in participants with type 2 diabetes at high risk for cardiovascular disease [1–6]. Furthermore, SGLT2 inhibitors were found to reduce the risk of renal disease progression by 45%, and show similar benefits for patients with and without established cardiovascular disease in a systematic review and meta-analysis [7]. Although a beneficial effect of SGLT2 inhibitors on the decrease in estimated glomerular filtration rate (eGFR) was observed in participants with type 2 diabetes and chronic kidney disease (CKD) [8], it remains unclear whether the renoprotective effects of SGLT2 inhibitors vary depending on the degree of albuminuria. In the present study, we compared the effects of SGLT2 inhibitors on renal function in participants with type 2 diabetes and CKD categorized into three groups: normoalbuminuria, microalbuminuria, and macroalbuminuria.

## Materials and methods

### Study design and participants

The analyzed data were obtained from our previous retrospective observational study [9]. We conducted a retrospective review of the clinical records of Japanese participants with type 2 diabetes (age > 20 years; SGLT2 inhibitor treatment > 2 years; eGFR < 60 mL/min/1.73 m^2^) in the outpatient center at Kurihara Clinic. Participants were excluded from the study if they had no clear data on eGFR or albuminuria. Participants with type 1 diabetes, and those with poor adherence or interruption of the medication were also excluded. An opt-out consent procedure was used. The study was conducted with approval from the Institutional Review Board of the Japan Clinicians Diabetes Association, and registered with the University Hospital Medical Information Network (UMIN; number UMIN000035263).

### Study definitions and outcomes

Based on the urinary albumin-to-creatinine ratio (UACR) or urinary protein-to-creatinine ratio (UPCR) at the start of SGLT2 inhibitor administration, participants were categorized into three groups: normoalbuminuria (A1; UACR < 30 mg/g Cr or UPCR < 0.15 g/g Cr), microalbuminuria (A2; UACR 30 to < 300 mg/g Cr or UPCR 0.15 to < 0.50 g/g Cr), and macroalbuminuria (A3; UACR ≥ 300 mg/g Cr or UPCR ≥ 0.50 g/g Cr) [10, 11]. The study outcome was a comparison of the rates of change in renal function evaluated by eGFR at 2 years after starting SGLT2 inhibitor (%∆eGFR + 2y) among the three groups. As previously described [9],  %∆eGFR + 2y and  %∆eGFR − 2y were calculated as follows:  %∆eGFR + 2y = [(eGFR at 2 years after starting SGLT2 inhibitor) − (eGFR at start of SGLT2 inhibitor)]/(eGFR at start of SGLT2 inhibitor);  %∆eGFR–2y = [(eGFR at start of SGLT2 inhibitor) − (eGFR at 2 years before starting SGLT2 inhibitor)]/(eGFR at 2 years before starting SGLT2 inhibitor).

### Statistical analysis

Data were expressed as mean ± standard deviation or median. Differences in baseline characteristics and changes among the three groups were compared by one-way analysis of variance followed by post hoc Bonferroni test or the Chi square test, as appropriate. Simple linear regression analyses were performed to test for associations between  %∆eGFR + 2y and baseline HbA1c or change in HbA1c. Values of *P* < 0.05 were considered to indicate statistical significance. All statistical analyses were performed using GraphPad Prism 7 (GraphPad Software, San Diego, CA, USA) and Microsoft Excel Statistics 2012 for Windows (SSRI Co. Ltd, Tokyo, Japan).

## Results

**Fig. 1:**
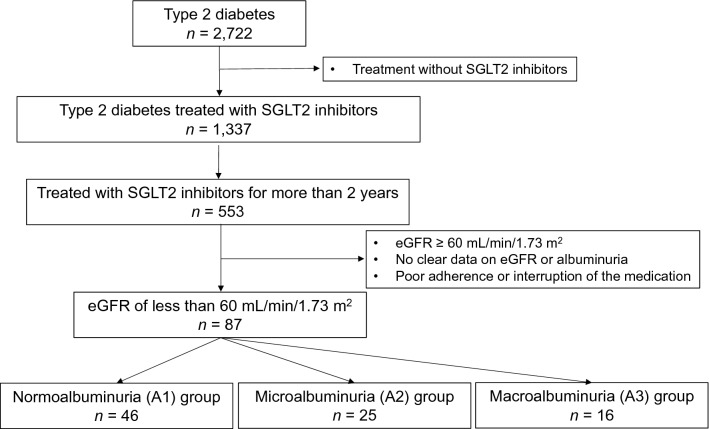
Flow diagram of the analysis in the study. *eGFR* estimated glomerular filtration rate, *SGLT2* sodium–glucose cotransporter 2

A total of 87 participants (40 females, 47 males) were divided into the three groups as follows (Fig. 1): A1 (*n* = 46), A2 (*n* = 25), and A3 (*n* = 16). The baseline characteristics of the participants are shown in Table 1. There were no significant differences among the three groups in most of the parameters, including sex, age, body mass index (BMI), duration of diabetes, eGFR, and blood pressure. However, HbA1c level in the A3 group was significantly higher than that in the A1 group.

**Table 1:** Baseline characteristics of the 87 participants

	A1	A2	A3	*P* value
*n*	46	25	16	
Sex (male/female)	21/25	16/9	10/6	0.251
Age (years)	67.0 ± 8.9	66.8 ± 7.0	63.3 ± 8.9	0.287
Body mass index (kg/m^2^)	29.8 ± 6.7	29.0 ± 4.2	28.3 ± 4.2	0.632
Body weight (kg)	75.5 ± 18.6	76.9 ± 13.4	74.0 ± 12.2	0.859
Duration of diabetes (years)	14.6 ± 6.7	15.8 ± 7.6	18.0 ± 8.8	0.284
HbA1c (%)	7.1 ± 0.7	7.2 ± 0.8	7.8 ± 1.0 *	0.015
eGFR (mL/min/1.73 m^2^)	47.2 ± 8.7	48.5 ± 8.8	42.5 ± 9.6	0.105
Category of GFR (G3a/G3b/G4)	27/18/1	18/6/1	9/6/1	0.681
Systolic blood pressure (mmHg)	127.6 ± 8.9	130.6 ± 11.5	131.2 ± 10.8	0.323
Diastolic blood pressure (mmHg)	74.3 ± 7.7	72.9 ± 10.6	73.8 ± 7.1	0.813
ARB or ACE-I (%)	82.6	96.0	100.0	
SGLT2 inhibitor (%)				
Canagliflozin	23.9	12.0	37.5	
Dapagliflozin	13.0	8.0	6.3	
Empagliflozin	8.7	4.0	12.5	
Ipragliflozin	4.3	20.0	18.8	
Luseogliflozin	6.5	20.0	6.3	
Tofogliflozin	43.5	36.0	18.8	
Other oral hypoglycemic agents (%)				
Sulfonylurea	50.0	52.0	62.5	
Biguanide	71.7	80.0	93.8	
Thiazolidinedione	2.2	8.0	14.3	
Alpha-glucosidase inhibitor	15.0	20.0	0.0	
Glinide	0.0	0.0	0.0	
DPP-4 inhibitor	50.0	76.0	62.5	
Insulin (%)	13.0	12.0	25.0	

Values are expressed as mean ± SD

*ACE-I* angiotensin-converting enzyme inhibitor, *ARB* angiotensin II receptor blocker, *DPP-4* dipeptidyl peptidase-4, *eGFR* estimated glomerular filtration rate, *SGLT2* sodium–glucose cotransporter 2

*P < 0.05 vs. A1 group

**Fig. 2:**
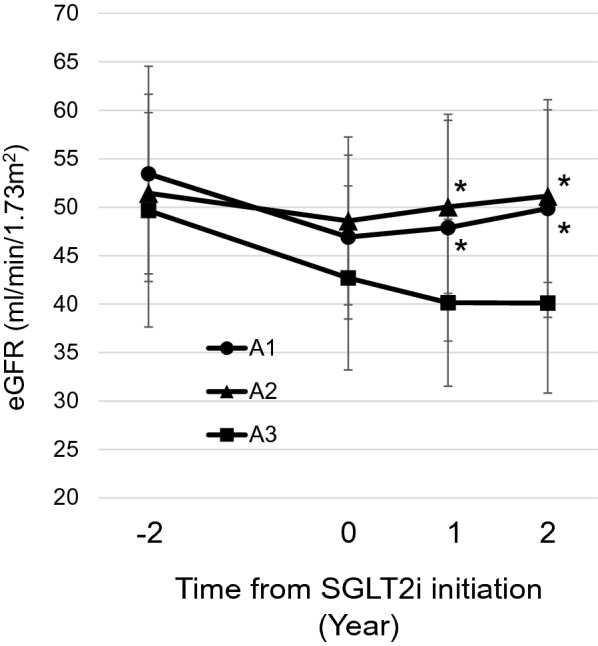
Serial changes in estimated glomerular filtration rate (eGFR) at 2 years before and after starting sodium–glucose cotransporter 2 (SGLT2) inhibitor in participants with type 2 diabetes and chronic kidney disease in three groups: normoalbuminuria (A1; *n* = 46), microalbuminuria (A2; *n* = 25), and macroalbuminuria (A3; *n* = 16). *P < 0.05 vs. A3, one-way analysis of variance followed by post hoc Bonferroni test

**Fig. 3:**
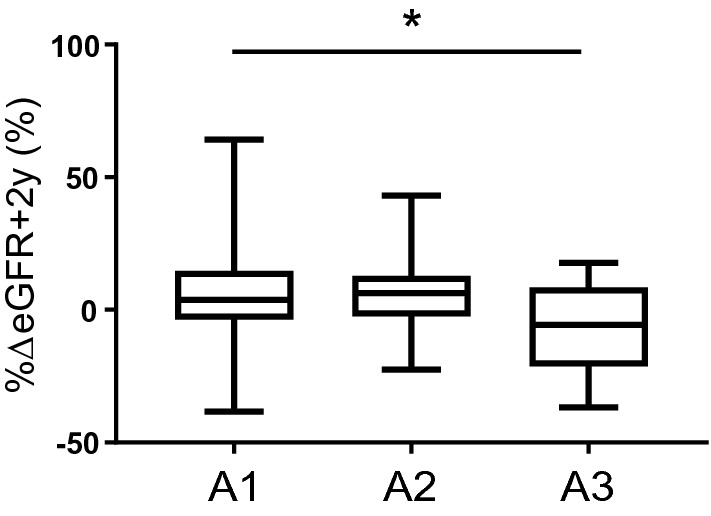
Rates of change in estimated glomerular filtration rate (eGFR) at 2 years after starting sodium–glucose cotransporter 2 (SGLT2) inhibitor (%∆eGFR + 2y) in participants with type 2 diabetes and chronic kidney disease in three groups: normoalbuminuria (A1; *n* = 46), microalbuminuria (A2; *n* = 25), and macroalbuminuria (A3; *n* = 16). *P < 0.05 between A1 and A3 group, one-way analysis of variance followed by post hoc Bonferroni test

As shown in Fig. 2, eGFR was similarly decreased at 2 years before starting SGLT2 inhibitor in all three groups (%∆eGFR − 2y: − 10.8% ± 13.5% in A1 group, − 5.1% ± 12.6% in A2 group, − 12.8 ± 14.7% in A3 group; *P* = 0.135). However, the decline in eGFR was ameliorated at 2 years after starting SGLT2 inhibitor, and eGFR was rather increased in the A1 and A2 groups. Interestingly,  %∆eGFR + 2y in the A1 group was significantly higher than that in the A3 group (Fig. 3).  %∆eGFR + 2y was not associated with baseline HbA1c (correlation coefficient: 0.068, *P* = 0.532) or change in HbA1c (correlation coefficient: − 0.101, *P* = 0.353).

## Discussion

The present results indicated that  %∆eGFR + 2y in the A1 group was significantly higher than that in the A3 group. Because baseline HbA1c in the A1 group was lower than that in the A3 group, we assumed that glucose tolerance could affect  %∆eGFR + 2y. However, there was no association between  %∆eGFR + 2y and baseline HbA1c or change in HbA1c at 2 years. These data suggest that glucose tolerance is not related to  %∆eGFR + 2y. Recently, it was reported that the incidence of low eGFR with normoalbuminuria has been increasing in type 2 diabetes [10, 12, 13]. Pathological findings revealed that tubulointerstitial and vascular lesions tended to be more advanced in participants with type 2 diabetes and CKD with normoalbuminuria than those in participants with microalbuminuria or macroalbuminuria [11, 14, 15]. Because the possible mechanisms for the renoprotective effects of SGLT2 inhibitors are assumed to include not only reduction in glomerular hyperfiltration as a result of tubuloglomerular feedback restoration, but also improvement of tubulointerstitial damage [16, 17], improved tubular cell injury may contribute to the greater beneficial effects of SGLT2 inhibitors in participants with type 2 diabetes and CKD with normoalbuminuria. Recent reports have shown a putative protective effect of SGLT2 inhibitors against tubular cell injury. In in vitro studies of proximal tubular cells, SGLT2 inhibitors suppressed the hyperglycemia-induced production of reactive oxygen species and angiotensinogen [18, 19]. In mice fed a high-fat diet, ipragliflozin improved proximal tubular cell integrity by reducing mitochondrial damage [20]. Furthermore, a cross-over clinical study showed that treatment with dapagliflozin reduced the urinary excretion of markers of tubular injury and inflammatory markers in participants with type 2 diabetes [17].

The main limitations of the present study are its retrospective design and relatively small sample. Other limitations are the relatively short follow-up duration and the study endpoint of changes in renal function evaluated by eGFR, rather than hard endpoints such as initiation of renal-replacement therapy or death from renal disease. Further studies are needed to verify our results in a prospective larger cohort for a longer observational period.

## Conclusions

In conclusion, our study demonstrated that more favorable effects of SGLT2 inhibitors on renal function were observed in participants with type 2 diabetes and CKD with normoalbuminuria than in participants with macroalbuminuria. These findings suggest that the renoprotective effects of SGLT2 inhibitors in participants with CKD can vary depending on the degree of albuminuria.

## Data Availability

The datasets used and/or analyzed during the current study are available from the corresponding author on reasonable request.

## Abbreviations

CKD: chronic kidney disease
eGFR: estimated glomerular filtration rate
SGLT2: sodium–glucose cotransporter 2
UACR: urinary albumin-to-creatinine ratio
UPCR: urinary protein-to-creatinine ratio
